# Crystal structure of dimethyl 3,3′-[(3-fluoro­phenyl)methyl­ene]bis­(1*H*-indole-2-carboxyl­ate)

**DOI:** 10.1107/S1600536814025756

**Published:** 2014-11-29

**Authors:** Xin-Hua Lu, Hong-Shun Sun, Jin Hu

**Affiliations:** a Department of Applied Chemistry, Nanjing College of Chemical Technology, Nanjing 210048, People’s Republic of China; b Chemical Engineering Department, Nanjing College of Chemical Technology, Nanjing 210048, People’s Republic of China

**Keywords:** crystal structure, indole, MRI contrast agent, N—H⋯O hydrogen bonds, C—H⋯π inter­actions

## Abstract

In the title compound, C_27_H_21_FN_2_O_4_, the mean planes of the two indole ring systems (r.m.s. deviations = 0.0166 and 0.0086 Å) are approximately perpendic­ular to one another, making a dihedral angle of 87.8 (5)°; the fluorobenzene ring is twisted with respect to the mean planes of the two indole ring systems at 82.7 (5) and 85.5 (3)°. In the crystal, pairs of N—H⋯O hydrogen bonds link the mol­ecules into the inversion dimers, which are further linked by N—H⋯O hydrogen bonds into supra­molecular chains propagating along the *b-*axis direction. Weak C—H⋯π inter­actions are observed between neighbouring chains.

## Abstract 2

In the title compound, the two indole ring systems are approximately perpendicular to one another, making a dihedral angle of 87.8 (5)°. In the crystal, pairs of N—H⋯O hydrogen bonds link the mol­ecules into the inversion dimers, which are further linked by N—H⋯O hydrogen bonds into supra­molecular chains propagating along the *b-*axis direction.

## Chemical context   

The indole unit forms the basis for general bis­(indoly)methanes, which are widely present in bioactive metabolites of numerous compounds isolated from natural sources (Poter *et al.*, 1977 ▶; Sundberg, 1996 ▶). In addition, bis­(indoly)methanes are important anti­biotics in the field of pharmaceuticals and the precursor of bioactive metabolites of terrestrial and marine origin (Chang *et al.*, 1999 ▶; Ge *et al.*, 1999 ▶). The title compound is one of the bis­(indoly)methane derivatives used as a precursor for MRI contrast agents (Ni, 2008 ▶). In recent years, we have reported the synthesis and crystal structures of some similar compounds (Sun *et al.*, 2012 ▶, 2013 ▶, 2014 ▶; Li *et al.*, 2014 ▶). Now we report herein on another bis­(indoly)methane compound.
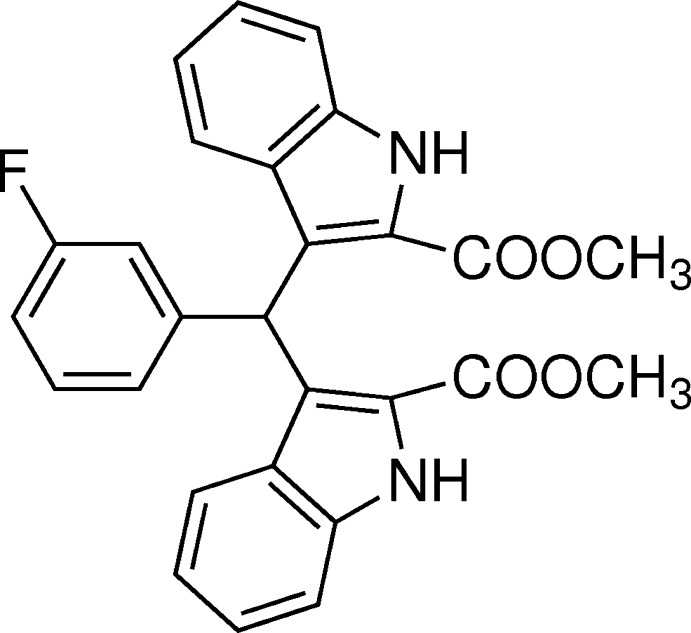


## Structural commentary   

**Figure 1:**
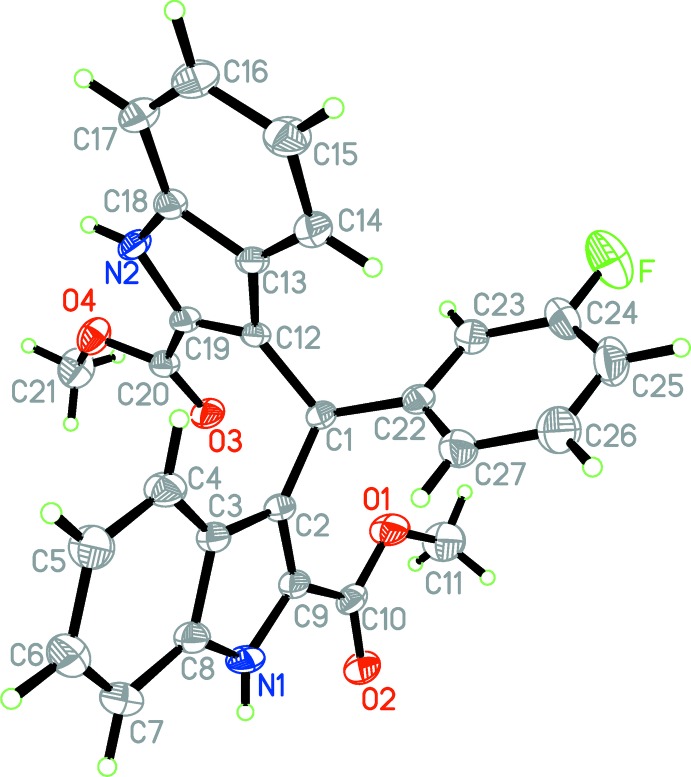
The mol­ecular structure of the title mol­ecule with the atom-labelling scheme. Displacement ellipsoids are drawn at the 30% probability level.

The mol­ecular structure of the title compound is shown in Fig. 1 ▶. The two indole ring systems are nearly perpendicular to each other [dihedral angle = 87.8 (5)°] while the benzene ring (C22–C27) is twisted to the N1/C2–C9 and N2/C12–C19 indole ring systems by dihedral angles of 82.7 (5) and 85.5 (3)°, respectively. The carboxyl groups are approximately co-planar with the attached indole ring systems, the dihedral angles between the carboxyl groups and the mean planes of the attached indole ring systems being 9.6 (3) and 9.6 (4)°.

## Supra­molecular features   

**Figure 2:**
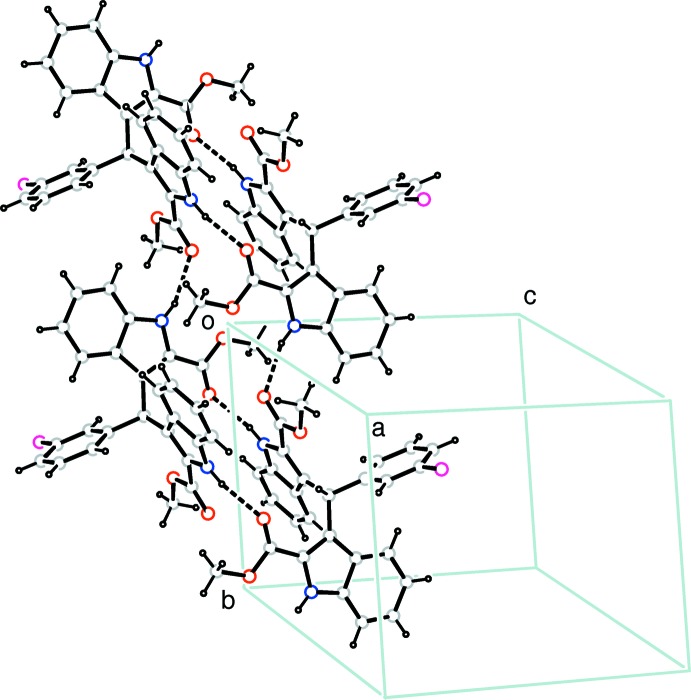
A packing diagram of the title compound. Hydrogen bonds are shown as dashed lines.

In the crystal, pairs of N1—H1*A*⋯O3^i^ [symmetry code: (i) −*x*, 1 − *y*, −*z*] hydrogen bonds link the mol­ecules into inversion dimmers, which are further linked by N2—H2*A*⋯O2^ii^ [symmetry code: (ii) *x*, 1 + *y*, *z*] hydrogen bonds into supra­molecular chains propagating along the *b*-axis direction (Table 1 ▶ and Fig. 2 ▶). Weak C—H⋯π inter­actions are also observed between neighbouring chains (Table 1 ▶).

## Database survey   

Several similar structures have been reported previously, *viz*. diethyl 3,3′-(phenyl­methyl­ene)bis­(1*H*-indole-2-carboxyl­ate) (Sun *et al.*, 2012 ▶), dimethyl 3,3′-(phenyl­methyl­ene)bis­(1*H*-indole-2-carboxyl­ate) (Sun *et al.*, 2013 ▶), dimethyl 3,3′-[(4-chloro­phen­yl) methyl­ene]bis­(1*H*-indole-2-carboxyl­ate) (Li *et al.*, 2014 ▶) and dimethyl 3,3′-[(3-nitro­phen­yl)methyl­ene]bis­(1*H*-indole-2-carboxyl­ate) ethanol monosolvate (Sun *et al.*, 2014 ▶). In those structures, the two indole ring systems are also nearly perpendicular to each other, the dihedral angles being 82.0 (5), 84.5 (5), 79.5 (4) and 89.3 (5)°, respectively.

## Synthesis and crystallization   

Methyl indole-2-carboxyl­ate (17.5 g, 100 mmol) was dissolved in 200 ml methanol; commercially available 3-fluoro­benz­aldehyde (6.2 g, 50 mmol) was added and the mixture was heated to reflux temperature. Concentrated HCl (3.7 ml) was added and the reaction was left for 1 h. After cooling, the white product was filtered off and washed thoroughly with methanol. The reaction was monitored by TLC (CHCl_3_:hexane = 1:1). The yield was 92%. Single crystals of the title compound suitable for X-ray analysis were obtained by slow evaporation of a methanol solution.

## Refinement   

Crystal data, data collection and structure refinement details are summarized in Table 2 ▶. H atoms were positioned geometrically with N—H = 0.86 and C—H = 0.93–0.98 Å, and constrained to ride on their parent atoms, with *U*_iso_(H) = *xU*_eq_(C,N), where *x* = 1.5 for methyl H atoms and 1.2 for the others.

## Supplementary Material

Crystal structure: contains datablock(s) I, global. DOI: 10.1107/S1600536814025756/xu5830sup1.cif

Structure factors: contains datablock(s) I. DOI: 10.1107/S1600536814025756/xu5830Isup2.hkl

Supporting information file. DOI: 10.1107/S1600536814025756/xu5830Isup3.cml

CCDC reference: 1036100

Additional supporting information:  crystallographic information; 3D view; checkCIF report

## Figures and Tables

**Table 1:** Hydrogen-bond geometry (, ) *Cg*4 is the centroid of the C13C18 ring.

*D*H*A*	*D*H	H*A*	*D* *A*	*D*H*A*
N1H1*A*O3^i^	0.86	2.06	2.913(3)	170
N2H2*A*O2^ii^	0.86	2.15	2.948(3)	155
C6H6*A* *Cg*4^iii^	0.93	2.75	3.645(4)	162

Symmetry codes: (i) 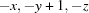; (ii) 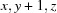; (iii) 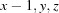.

**Table 2:** Experimental details

Crystal data	
Chemical formula	C_27_H_21_FN_2_O_4_
*M*_r_	456.46
Crystal system, space group	Triclinic, *P* 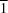
Temperature (K)	293
*a*, *b*, *c* ()	9.6980(19), 10.119(2), 12.875(3)
, , ()	89.86(3), 83.10(3), 65.45(3)
*V* (^3^)	1139.4(4)
*Z*	2
Radiation type	Mo *K*
(mm^1^)	0.10
Crystal size (mm)	0.30 0.20 0.10

Data collection	
Diffractometer	EnrafNonius CAD-4
Absorption correction	scan (North *et al.*, 1968 ▶)
*T*_min_, *T*_max_	0.972, 0.991
No. of measured, independent and observed [*I* > 2(*I*)] reflections	4453, 4183, 2587
*R*_int_	0.036
(sin /)_max_ (^1^)	0.603

Refinement	
*R*[*F* ^2^ > 2(*F* ^2^)], *wR*(*F* ^2^), *S*	0.060, 0.163, 1.00
No. of reflections	4183
No. of parameters	307
No. of restraints	1
H-atom treatment	H-atom parameters constrained
_max_, _min_ (e ^3^)	0.72, 0.25

Computer programs: *CAD-4 EXPRESS* (EnrafNonius, 1994 ▶), *XCAD4* (Harms Wocadlo, 1995 ▶) and *SHELXTL* (Sheldrick, 2008 ▶).

## supplementary crystallographic information

### Crystal data

**Table:**

C_27_H_21_FN_2_O_4_	*Z* = 2
*M**_r_* = 456.46	*F*(000) = 476
Triclinic, *P*1	*D*_x_ = 1.331 Mg m^−^^3^
Hall symbol: -P 1	Mo *K*α radiation, λ = 0.71073 Å
*a* = 9.6980 (19) Å	Cell parameters from 25 reflections
*b* = 10.119 (2) Å	θ = 9–13°
*c* = 12.875 (3) Å	µ = 0.10 mm^−^^1^
α = 89.86 (3)°	*T* = 293 K
β = 83.10 (3)°	Block, colorless
γ = 65.45 (3)°	0.30 × 0.20 × 0.10 mm
*V* = 1139.4 (4) Å^3^	

### Data collection

**Table:**

Enraf–Nonius CAD-4 diffractometer	2587 reflections with *I* > 2σ(*I*)
Radiation source: fine-focus sealed tube	*R*_int_ = 0.036
Graphite monochromator	θ_max_ = 25.4°, θ_min_ = 1.6°
ω/2θ scans	*h* = 0→11
Absorption correction: ψ scan (North *et al.*, 1968)	*k* = −11→12
*T*_min_ = 0.972, *T*_max_ = 0.991	*l* = −15→15
4453 measured reflections	3 standard reflections every 200 reflections
4183 independent reflections	intensity decay: 1%

### Refinement

**Table:**

Refinement on *F*^2^	Primary atom site location: structure-invariant direct methods
Least-squares matrix: full	Secondary atom site location: difference Fourier map
*R*[*F*^2^ > 2σ(*F*^2^)] = 0.060	Hydrogen site location: inferred from neighbouring sites
*wR*(*F*^2^) = 0.163	H-atom parameters constrained
*S* = 1.00	*w* = 1/[σ^2^(*F*_o_^2^) + (0.085*P*)^2^] where *P* = (*F*_o_^2^ + 2*F*_c_^2^)/3
4183 reflections	(Δ/σ)_max_ < 0.001
307 parameters	Δρ_max_ = 0.72 e Å^−^^3^
1 restraint	Δρ_min_ = −0.25 e Å^−^^3^

### Special details

**Table:**

Geometry. All e.s.d.'s (except the e.s.d. in the dihedral angle between two l.s. planes) are estimated using the full covariance matrix. The cell e.s.d.'s are taken into account individually in the estimation of e.s.d.'s in distances, angles and torsion angles; correlations between e.s.d.'s in cell parameters are only used when they are defined by crystal symmetry. An approximate (isotropic) treatment of cell e.s.d.'s is used for estimating e.s.d.'s involving l.s. planes.
Refinement. Refinement of *F*^2^ against ALL reflections. The weighted *R*-factor *wR* and goodness of fit *S* are based on *F*^2^, conventional *R*-factors *R* are based on *F*, with *F* set to zero for negative *F*^2^. The threshold expression of *F*^2^ > σ(*F*^2^) is used only for calculating *R*-factors(gt) *etc*. and is not relevant to the choice of reflections for refinement. *R*-factors based on *F*^2^ are statistically about twice as large as those based on *F*, and *R*- factors based on ALL data will be even larger.

### Fractional atomic coordinates and isotropic or equivalent isotropic displacement parameters (Å^2^)

**Table:**

	*x*	*y*	*z*	*U*_iso_*/*U*_eq_	
N1	−0.1052 (3)	0.5029 (2)	0.13830 (18)	0.0445 (6)	
H1A	−0.1408	0.4496	0.1090	0.053*	
O1	0.3009 (2)	0.3113 (2)	0.08820 (18)	0.0586 (6)	
C1	0.2144 (3)	0.5855 (3)	0.2059 (2)	0.0334 (6)	
H1B	0.2859	0.5329	0.1441	0.040*	
O2	0.1188 (3)	0.2409 (2)	0.05471 (17)	0.0555 (6)	
N2	0.1837 (3)	0.9546 (2)	0.14191 (17)	0.0396 (6)	
H2A	0.1767	1.0211	0.0985	0.048*	
C2	0.0646 (3)	0.5803 (3)	0.1908 (2)	0.0341 (6)	
O3	0.2455 (2)	0.6468 (2)	−0.02476 (15)	0.0486 (5)	
C3	−0.0884 (3)	0.6830 (3)	0.2299 (2)	0.0359 (6)	
O4	0.1821 (3)	0.8813 (2)	−0.05567 (15)	0.0529 (6)	
C4	−0.1512 (3)	0.8120 (3)	0.2943 (2)	0.0439 (7)	
H4A	−0.0880	0.8485	0.3207	0.053*	
F	0.6593 (3)	0.3395 (3)	0.38335 (19)	0.1028 (8)	
C5	−0.3065 (4)	0.8827 (3)	0.3173 (2)	0.0522 (8)	
H5B	−0.3483	0.9676	0.3601	0.063*	
C6	−0.4039 (4)	0.8314 (3)	0.2787 (3)	0.0545 (8)	
H6A	−0.5090	0.8841	0.2951	0.065*	
C7	−0.3497 (4)	0.7059 (3)	0.2175 (3)	0.0518 (8)	
H7A	−0.4154	0.6715	0.1924	0.062*	
C8	−0.1906 (3)	0.6313 (3)	0.1940 (2)	0.0394 (7)	
C9	0.0469 (3)	0.4723 (3)	0.1365 (2)	0.0368 (6)	
C10	0.1559 (4)	0.3315 (3)	0.0883 (2)	0.0416 (7)	
C11	0.4154 (4)	0.1713 (4)	0.0498 (3)	0.0767 (11)	
H11A	0.5149	0.1686	0.0529	0.115*	
H11B	0.4049	0.1537	−0.0215	0.115*	
H11C	0.4031	0.0977	0.0921	0.115*	
C12	0.2062 (3)	0.7399 (3)	0.2047 (2)	0.0312 (6)	
C13	0.1975 (3)	0.8347 (3)	0.2903 (2)	0.0331 (6)	
C14	0.2017 (3)	0.8230 (3)	0.3985 (2)	0.0416 (7)	
H14A	0.2120	0.7368	0.4295	0.050*	
C15	0.1906 (4)	0.9398 (3)	0.4581 (2)	0.0517 (8)	
H15A	0.1949	0.9317	0.5297	0.062*	
C16	0.1728 (4)	1.0709 (3)	0.4134 (3)	0.0547 (9)	
H16A	0.1632	1.1488	0.4563	0.066*	
C17	0.1692 (4)	1.0876 (3)	0.3093 (2)	0.0489 (8)	
H17A	0.1586	1.1748	0.2799	0.059*	
C18	0.1821 (3)	0.9681 (3)	0.2474 (2)	0.0373 (7)	
C19	0.1986 (3)	0.8161 (3)	0.1163 (2)	0.0339 (6)	
C20	0.2100 (3)	0.7701 (3)	0.0063 (2)	0.0376 (7)	
C21	0.1995 (4)	0.8471 (4)	−0.1662 (2)	0.0666 (10)	
H21A	0.1770	0.9343	−0.2036	0.100*	
H21B	0.1303	0.8053	−0.1797	0.100*	
H21C	0.3027	0.7788	−0.1891	0.100*	
C22	0.2846 (3)	0.5040 (3)	0.2992 (2)	0.0367 (7)	
C23	0.4402 (3)	0.4571 (3)	0.3005 (2)	0.0449 (7)	
H23A	0.5000	0.4746	0.2449	0.054*	
C24	0.5055 (4)	0.3846 (4)	0.3845 (3)	0.0548 (8)	
C25	0.4234 (4)	0.3559 (4)	0.4673 (3)	0.0629 (10)	
H25A	0.4706	0.3062	0.5232	0.076*	
C26	0.2694 (4)	0.4020 (4)	0.4664 (3)	0.0639 (10)	
H26A	0.2105	0.3844	0.5226	0.077*	
C27	0.2007 (4)	0.4745 (3)	0.3822 (2)	0.0491 (8)	
H27A	0.0963	0.5036	0.3820	0.059*	

### Atomic displacement parameters (Å^2^)

**Table:**

	*U*^11^	*U*^22^	*U*^33^	*U*^12^	*U*^13^	*U*^23^
N1	0.0524 (17)	0.0385 (13)	0.0546 (15)	−0.0295 (12)	−0.0120 (12)	−0.0062 (11)
O1	0.0498 (14)	0.0351 (11)	0.0876 (17)	−0.0160 (10)	−0.0029 (12)	−0.0176 (11)
C1	0.0389 (16)	0.0255 (13)	0.0378 (15)	−0.0145 (12)	−0.0079 (12)	−0.0016 (11)
O2	0.0730 (16)	0.0339 (11)	0.0677 (14)	−0.0277 (11)	−0.0187 (12)	−0.0071 (10)
N2	0.0548 (16)	0.0283 (11)	0.0403 (14)	−0.0212 (11)	−0.0092 (11)	0.0015 (10)
C2	0.0378 (16)	0.0297 (13)	0.0389 (15)	−0.0173 (12)	−0.0088 (12)	−0.0001 (11)
O3	0.0655 (15)	0.0392 (11)	0.0468 (12)	−0.0257 (10)	−0.0143 (10)	−0.0053 (9)
C3	0.0392 (16)	0.0349 (14)	0.0392 (15)	−0.0200 (13)	−0.0086 (12)	0.0029 (12)
O4	0.0744 (16)	0.0425 (11)	0.0401 (12)	−0.0228 (11)	−0.0075 (10)	0.0044 (9)
C4	0.0446 (19)	0.0390 (16)	0.0507 (18)	−0.0197 (14)	−0.0065 (14)	−0.0108 (14)
F	0.0659 (15)	0.131 (2)	0.0963 (18)	−0.0211 (15)	−0.0317 (13)	0.0148 (15)
C5	0.0440 (19)	0.0439 (17)	0.063 (2)	−0.0142 (15)	0.0002 (15)	−0.0118 (15)
C6	0.0383 (19)	0.055 (2)	0.067 (2)	−0.0171 (16)	−0.0064 (16)	0.0020 (17)
C7	0.0432 (19)	0.0553 (19)	0.067 (2)	−0.0292 (16)	−0.0115 (16)	0.0039 (16)
C8	0.0422 (17)	0.0407 (16)	0.0422 (16)	−0.0233 (14)	−0.0094 (13)	0.0029 (13)
C9	0.0433 (17)	0.0332 (14)	0.0400 (16)	−0.0205 (13)	−0.0111 (13)	0.0015 (12)
C10	0.057 (2)	0.0320 (14)	0.0411 (16)	−0.0232 (14)	−0.0096 (14)	0.0026 (12)
C11	0.058 (2)	0.047 (2)	0.108 (3)	−0.0091 (18)	0.003 (2)	−0.020 (2)
C12	0.0289 (14)	0.0286 (13)	0.0399 (15)	−0.0149 (11)	−0.0085 (12)	−0.0012 (11)
C13	0.0305 (15)	0.0307 (14)	0.0413 (16)	−0.0156 (12)	−0.0066 (12)	−0.0027 (12)
C14	0.0461 (18)	0.0370 (15)	0.0437 (17)	−0.0187 (14)	−0.0076 (13)	0.0002 (13)
C15	0.064 (2)	0.0522 (19)	0.0421 (17)	−0.0270 (17)	−0.0089 (15)	−0.0080 (15)
C16	0.067 (2)	0.0413 (17)	0.055 (2)	−0.0216 (16)	−0.0093 (16)	−0.0156 (15)
C17	0.056 (2)	0.0346 (15)	0.058 (2)	−0.0204 (15)	−0.0095 (15)	−0.0068 (14)
C18	0.0351 (16)	0.0317 (14)	0.0451 (17)	−0.0133 (12)	−0.0080 (13)	−0.0043 (12)
C19	0.0376 (16)	0.0297 (13)	0.0384 (15)	−0.0174 (12)	−0.0070 (12)	−0.0028 (12)
C20	0.0383 (17)	0.0363 (15)	0.0414 (16)	−0.0180 (13)	−0.0078 (13)	−0.0015 (13)
C21	0.088 (3)	0.069 (2)	0.0409 (19)	−0.031 (2)	−0.0089 (18)	0.0063 (16)
C22	0.0415 (17)	0.0274 (13)	0.0429 (16)	−0.0155 (12)	−0.0075 (13)	−0.0046 (12)
C23	0.0471 (19)	0.0438 (16)	0.0451 (18)	−0.0196 (14)	−0.0083 (14)	0.0030 (14)
C24	0.0472 (19)	0.0567 (19)	0.054 (2)	−0.0121 (16)	−0.0195 (16)	0.0022 (16)
C25	0.078 (3)	0.062 (2)	0.048 (2)	−0.023 (2)	−0.0242 (19)	0.0152 (16)
C26	0.075 (3)	0.072 (2)	0.052 (2)	−0.037 (2)	−0.0126 (18)	0.0107 (18)
C27	0.0469 (19)	0.0564 (19)	0.0498 (19)	−0.0263 (16)	−0.0105 (15)	0.0107 (15)

### Geometric parameters (Å, º)

**Table:**

N1—C8	1.362 (3)	C11—H11A	0.9600
N1—C9	1.373 (4)	C11—H11B	0.9600
N1—H1A	0.8600	C11—H11C	0.9600
O1—C10	1.333 (4)	C12—C19	1.365 (4)
O1—C11	1.431 (4)	C12—C13	1.435 (3)
C1—C2	1.511 (4)	C13—C14	1.402 (4)
C1—C22	1.520 (4)	C13—C18	1.413 (4)
C1—C12	1.532 (3)	C14—C15	1.370 (4)
C1—H1B	0.9800	C14—H14A	0.9300
O2—C10	1.213 (3)	C15—C16	1.396 (4)
N2—C18	1.363 (3)	C15—H15A	0.9300
N2—C19	1.385 (3)	C16—C17	1.353 (4)
N2—H2A	0.8600	C16—H16A	0.9300
C2—C9	1.379 (3)	C17—C18	1.403 (4)
C2—C3	1.440 (4)	C17—H17A	0.9300
O3—C20	1.203 (3)	C19—C20	1.469 (4)
C3—C4	1.409 (4)	C21—H21A	0.9600
C3—C8	1.419 (4)	C21—H21B	0.9600
O4—C20	1.330 (3)	C21—H21C	0.9600
O4—C21	1.439 (3)	C22—C27	1.373 (4)
C4—C5	1.366 (4)	C22—C23	1.383 (4)
C4—H4A	0.9300	C23—C24	1.372 (4)
F—C24	1.364 (4)	C23—H23A	0.9300
C5—C6	1.389 (4)	C24—C25	1.357 (5)
C5—H5B	0.9300	C25—C26	1.370 (5)
C6—C7	1.364 (4)	C25—H25A	0.9300
C6—H6A	0.9300	C26—C27	1.385 (4)
C7—C8	1.401 (4)	C26—H26A	0.9300
C7—H7A	0.9300	C27—H27A	0.9300
C9—C10	1.458 (4)		
			
C8—N1—C9	109.0 (2)	C13—C12—C1	129.4 (2)
C8—N1—H1A	125.5	C14—C13—C18	118.0 (2)
C9—N1—H1A	125.5	C14—C13—C12	135.4 (2)
C10—O1—C11	116.3 (2)	C18—C13—C12	106.6 (2)
C2—C1—C22	113.7 (2)	C15—C14—C13	119.3 (3)
C2—C1—C12	112.9 (2)	C15—C14—H14A	120.4
C22—C1—C12	112.7 (2)	C13—C14—H14A	120.4
C2—C1—H1B	105.5	C14—C15—C16	121.3 (3)
C22—C1—H1B	105.5	C14—C15—H15A	119.4
C12—C1—H1B	105.5	C16—C15—H15A	119.4
C18—N2—C19	108.7 (2)	C17—C16—C15	121.8 (3)
C18—N2—H2A	125.7	C17—C16—H16A	119.1
C19—N2—H2A	125.7	C15—C16—H16A	119.1
C9—C2—C3	105.3 (2)	C16—C17—C18	117.3 (3)
C9—C2—C1	126.3 (2)	C16—C17—H17A	121.3
C3—C2—C1	128.5 (2)	C18—C17—H17A	121.3
C4—C3—C8	117.9 (3)	N2—C18—C17	129.5 (3)
C4—C3—C2	134.8 (3)	N2—C18—C13	108.2 (2)
C8—C3—C2	107.3 (2)	C17—C18—C13	122.3 (3)
C20—O4—C21	116.2 (2)	C12—C19—N2	109.8 (2)
C5—C4—C3	118.9 (3)	C12—C19—C20	130.1 (2)
C5—C4—H4A	120.6	N2—C19—C20	120.1 (2)
C3—C4—H4A	120.6	O3—C20—O4	124.0 (2)
C4—C5—C6	121.9 (3)	O3—C20—C19	124.5 (3)
C4—C5—H5B	119.0	O4—C20—C19	111.4 (2)
C6—C5—H5B	119.0	O4—C21—H21A	109.5
C7—C6—C5	121.8 (3)	O4—C21—H21B	109.5
C7—C6—H6A	119.1	H21A—C21—H21B	109.5
C5—C6—H6A	119.1	O4—C21—H21C	109.5
C6—C7—C8	117.0 (3)	H21A—C21—H21C	109.5
C6—C7—H7A	121.5	H21B—C21—H21C	109.5
C8—C7—H7A	121.5	C27—C22—C23	118.4 (3)
N1—C8—C7	129.8 (3)	C27—C22—C1	123.0 (3)
N1—C8—C3	107.7 (2)	C23—C22—C1	118.5 (3)
C7—C8—C3	122.5 (3)	C24—C23—C22	119.4 (3)
N1—C9—C2	110.7 (2)	C24—C23—H23A	120.3
N1—C9—C10	116.5 (2)	C22—C23—H23A	120.3
C2—C9—C10	132.5 (3)	C25—C24—F	119.4 (3)
O2—C10—O1	123.6 (3)	C25—C24—C23	122.6 (3)
O2—C10—C9	123.6 (3)	F—C24—C23	118.0 (3)
O1—C10—C9	112.8 (2)	C24—C25—C26	118.2 (3)
O1—C11—H11A	109.5	C24—C25—H25A	120.9
O1—C11—H11B	109.5	C26—C25—H25A	120.9
H11A—C11—H11B	109.5	C25—C26—C27	120.4 (3)
O1—C11—H11C	109.5	C25—C26—H26A	119.8
H11A—C11—H11C	109.5	C27—C26—H26A	119.8
H11B—C11—H11C	109.5	C22—C27—C26	121.0 (3)
C19—C12—C13	106.8 (2)	C22—C27—H27A	119.5
C19—C12—C1	123.7 (2)	C26—C27—H27A	119.5
			
C22—C1—C2—C9	−86.0 (3)	C1—C12—C13—C18	−176.2 (3)
C12—C1—C2—C9	143.9 (3)	C18—C13—C14—C15	0.1 (4)
C22—C1—C2—C3	93.1 (3)	C12—C13—C14—C15	179.7 (3)
C12—C1—C2—C3	−37.0 (4)	C13—C14—C15—C16	1.0 (5)
C9—C2—C3—C4	176.4 (3)	C14—C15—C16—C17	−1.5 (5)
C1—C2—C3—C4	−2.8 (5)	C15—C16—C17—C18	0.8 (5)
C9—C2—C3—C8	−1.3 (3)	C19—N2—C18—C17	180.0 (3)
C1—C2—C3—C8	179.4 (2)	C19—N2—C18—C13	0.5 (3)
C8—C3—C4—C5	−1.4 (4)	C16—C17—C18—N2	−179.1 (3)
C2—C3—C4—C5	−178.9 (3)	C16—C17—C18—C13	0.3 (4)
C3—C4—C5—C6	−0.3 (5)	C14—C13—C18—N2	178.7 (2)
C4—C5—C6—C7	1.4 (5)	C12—C13—C18—N2	−0.9 (3)
C5—C6—C7—C8	−0.7 (5)	C14—C13—C18—C17	−0.8 (4)
C9—N1—C8—C7	−179.6 (3)	C12—C13—C18—C17	179.6 (3)
C9—N1—C8—C3	−0.7 (3)	C13—C12—C19—N2	−0.7 (3)
C6—C7—C8—N1	177.7 (3)	C1—C12—C19—N2	176.7 (2)
C6—C7—C8—C3	−1.1 (4)	C13—C12—C19—C20	176.5 (3)
C4—C3—C8—N1	−176.9 (2)	C1—C12—C19—C20	−6.1 (4)
C2—C3—C8—N1	1.3 (3)	C18—N2—C19—C12	0.1 (3)
C4—C3—C8—C7	2.1 (4)	C18—N2—C19—C20	−177.4 (2)
C2—C3—C8—C7	−179.7 (3)	C21—O4—C20—O3	−1.2 (4)
C8—N1—C9—C2	−0.2 (3)	C21—O4—C20—C19	176.3 (3)
C8—N1—C9—C10	174.5 (2)	C12—C19—C20—O3	−8.5 (5)
C3—C2—C9—N1	0.9 (3)	N2—C19—C20—O3	168.5 (3)
C1—C2—C9—N1	−179.8 (2)	C12—C19—C20—O4	174.0 (3)
C3—C2—C9—C10	−172.5 (3)	N2—C19—C20—O4	−9.0 (4)
C1—C2—C9—C10	6.7 (5)	C2—C1—C22—C27	−22.3 (4)
C11—O1—C10—O2	−2.7 (4)	C12—C1—C22—C27	107.9 (3)
C11—O1—C10—C9	175.3 (3)	C2—C1—C22—C23	157.6 (2)
N1—C9—C10—O2	−3.0 (4)	C12—C1—C22—C23	−72.3 (3)
C2—C9—C10—O2	170.1 (3)	C27—C22—C23—C24	−0.8 (4)
N1—C9—C10—O1	179.0 (2)	C1—C22—C23—C24	179.4 (2)
C2—C9—C10—O1	−7.9 (4)	C22—C23—C24—C25	0.3 (5)
C2—C1—C12—C19	−72.7 (3)	C22—C23—C24—F	179.9 (3)
C22—C1—C12—C19	156.8 (2)	F—C24—C25—C26	−179.8 (3)
C2—C1—C12—C13	104.0 (3)	C23—C24—C25—C26	−0.2 (5)
C22—C1—C12—C13	−26.5 (4)	C24—C25—C26—C27	0.7 (5)
C19—C12—C13—C14	−178.6 (3)	C23—C22—C27—C26	1.2 (4)
C1—C12—C13—C14	4.3 (5)	C1—C22—C27—C26	−178.9 (3)
C19—C12—C13—C18	1.0 (3)	C25—C26—C27—C22	−1.2 (5)

### Hydrogen-bond geometry (Å, º)

Cg4 is the centroid of the C13–C18 ring.

**Table:**

*D*—H···*A*	*D*—H	H···*A*	*D*···*A*	*D*—H···*A*
N1—H1*A*···O3^i^	0.86	2.06	2.913 (3)	170
N2—H2*A*···O2^ii^	0.86	2.15	2.948 (3)	155
C6—H6*A*···*Cg*4^iii^	0.93	2.75	3.645 (4)	162

Symmetry codes: (i) −*x*, −*y*+1, −*z*; (ii) *x*, *y*+1, *z*; (iii) *x*−1, *y*, *z*.
